# From a Demand-Based to a Supply-Limited Framework of Brain Metabolism

**DOI:** 10.3389/fnint.2022.818685

**Published:** 2022-04-01

**Authors:** Suzana Herculano-Houzel, Douglas L. Rothman

**Affiliations:** ^1^Department of Psychology, Vanderbilt University, Nashville, TN, United States; ^2^Department of Biological Sciences, Vanderbilt University, Nashville, TN, United States; ^3^Vanderbilt Brain Institute, Vanderbilt University, Nashville, TN, United States; ^4^Department of Radiology and Biomedical Imaging, Yale University, New Haven, CT, United States; ^5^Department of Biomedical Engineering, Yale University, New Haven, CT, United States; ^6^Magnetic Resonance Research Center, Yale University, New Haven, CT, United States

**Keywords:** blood flow, brain metabolism, fMRI, neurovascular uncoupling, oxygen extraction factor, capillary density

## Abstract

What defines the rate of energy use by the brain, as well as per neurons of different sizes in different structures and animals, is one fundamental aspect of neuroscience for which much has been theorized, but very little data are available. The prevalent theories and models consider that energy supply from the vascular system to different brain regions is adjusted both dynamically and in the course of development and evolution to meet the *demands* of neuronal activity. In this perspective, we offer an alternative view: that regional rates of energy use might be mostly constrained by *supply*, given the properties of the brain capillary network, the highly stable rate of oxygen delivery to the whole brain under physiological conditions, and homeostatic constraints. We present evidence that these constraints, based on capillary density and tissue oxygen homeostasis, are similar between brain regions and mammalian species, suggesting they derive from fundamental biophysical limitations. The same constraints also determine the relationship between regional rates of brain oxygen supply and usage over the full physiological range of brain activity, from deep sleep to intense sensory stimulation, during which the apparent uncoupling of blood flow and oxygen use is still a predicted consequence of supply limitation. By carefully separating “energy cost” into energy supply and energy use, and doing away with the problematic concept of energetic “demands,” our new framework should help shine a new light on the neurovascular bases of metabolic support of brain function and brain functional imaging. We speculate that the trade-offs between functional systems and even the limitation to a single attentional spot at a time might be consequences of a strongly supply-limited brain economy. We propose that a deeper understanding of brain energy supply constraints will provide a new evolutionary understanding of constraints on brain function due to energetics; offer new diagnostic insight to disturbances of brain metabolism; lead to clear, testable predictions on the scaling of brain metabolic cost and the evolution of brains of different sizes; and open new lines of investigation into the microvascular bases of progressive cognitive loss in normal aging as well as metabolic diseases.

## Introduction

### The Various Dimensions of the Problem

The absolute and relative energy costs of the brain are highly variable across species. What determines how much energy the brain of a given species uses? In the human body, the brain is second only to the liver as the most energy-consuming organ, in absolute energetic cost per day (Aschoff et al., 1971). The high cost of the brain is not a reflection of its relative size in the body: The brain alone is estimated to use 8% of the total energy used by the whole body per day in mice, 13% of that energy in monkey, and as much as 20 or 25% in humans (Mink et al., 1981), even though it only represents 2-3% of body mass in all three species. Furthermore, it is well established that different brain areas have different metabolic rates even at rest. Is that because of different neuronal function, or because of structural differences that affect energy distribution, such as local differences in distribution of neurons, or synapses? Finally, brain function varies over time, and within a species, the regional rate of brain glucose metabolism can range from half the resting awake state in deep sleep to almost 50% higher during sensory stimulation. What determines this dynamic range of energy utilization rate? Surprisingly, despite much advance in neuroimaging methods and accomplishments, such fundamental aspects of brain functional organization and metabolism remain controversial.

Across species, the rate of brain glucose use at rest scales as a power function of brain mass with exponent 0.87, which is faster than the scaling of whole body metabolic rate with body mass raised to an exponent between 2/3 and 3/4 (Herculano-Houzel, 2011). The resting rate of glucose use across species is described even better as a linear function of the absolute number of neurons, with the energy cost for rodent and primate species (including humans) linearly proportional to its number of neurons (Herculano-Houzel, 2011). Because neuronal density in the brain does not scale universally with brain mass across mammals (Herculano-Houzel, 2016), it follows that the apparent non-linear scaling between brain energy use and brain mass may be a simple consequence of varying average neuronal density in the brain, and its exact exponent depends on the species sampled (Herculano-Houzel, 2011). It remains a limitation that functionally relevant measurements of whole brain energy use are available for only six species [mouse, rat, squirrel, monkey, baboon and human (Karbowski, 2007)].

*Within* a given brain, at rest (that is, without structured sensory stimulation), it also remains unclear what determines local rates of energy use, as different parts of the same brain are known to use energy at different rates. For example, of the energy that supports a given brain, about half is used by the cerebral cortex alone (Karbowski, 2007), even though this structure only contains between 15 and 25% of all brain neurons in mammals, while it corresponds to between around 40 and 80% of brain mass across species (Herculano-Houzel, 2016). Rates are typically highest in the inferior colliculus, higher in the neocortical gray matter than in the forebrain and hippocampus, and lowest in the white matter, in various species, and rates of glucose use, oxygen use, and blood flow are tightly coupled across sites [macaque (Kennedy et al., 1978); rabbit (Passero et al., 1981); cat (Chugani et al., 1991); mouse (Bouilleret et al., 2000); rat (Levant and Pazdernik, 2004)].

Different sites and structures in a brain are clearly built of different types and numbers of neurons with different cell sizes, function and firing rate frequencies, and this may explain regional differences in energy requirements. Alternatively, site-specific differences in resting metabolic rate could be imposed by site-specific capillary density and blood flow capacity.

Regional metabolic rates also vary over time, with what is a seemingly disproportionate increase in regional rates of cerebral blood flow (rCBF) versus regional rates of oxygen use (rCMRO_2_), termed “neurovascular uncoupling” [reviewed in Leithner and Royl (2014)]. This term refers to how, within a given brain site, the rates of cerebral blood flow (rCBF) increase up to 2-fold during sensory stimulation and focused mental activity, but that occurs with only approximately half the fractional increase in cerebral oxygen metabolism [rCMRO_2_; (Buxton, 2010; see Table 1 in Leithner and Royl, 2014)]. The discrepancy cannot be attributed to large changes in capillary recruitment (opening of previously closed capillaries) in the brain; while capillary recruitment underlies the massive increase in blood flow and energy use in skeletal muscle during exercise, it was ruled out in the brain after direct observation of red blood cell transit through the capillary bed (Kuschinsky and Paulson, 1992). Indeed, the perfused capillary network is similar across sleep and quiet waking states (Zoccoli et al., 1996), and red blood cell transit times have been shown to be relatively uniform across resting awake and stimulated conditions (Jesperson and Ostergaard, 2012).

**FIGURE 1:**
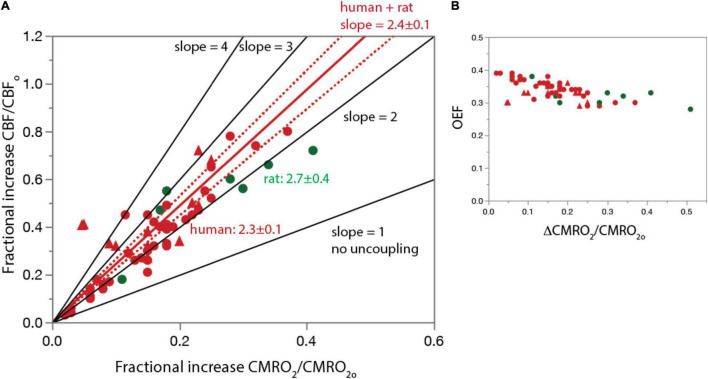
The basis for proposing neurovascular uncoupling: rCBF increases in excess of rCMRO_2_ and OEF drops upon structured stimulation. **(A)** Graph shows fractional increase in rCBF (ΔCBF/CBF_o_) plotted as a function of the fractional increase in rCMRO_2_ (ΔCMRO_2_/CMRO_2o_) in the human and rat visual cortex during photic stimulation. For comparison, lines are plotted with the predicted slope of 1 if there were no uncoupling, as well as higher slopes of 3 or 4. Human and rat data are equally well fit separately (not shown) and combined (plotted in red; dotted lines, 95% confidence interval) by linear functions of slope slightly above 2. Figure 4A is based on Figure 1 of Buxton (2010) but with additional data points. **(B)** OEF calculated from the same data points in A. Additional human data from Takahashi (1989), Madsen et al. (1991), Boyle et al. (1994), Marrett and Gjedde (1997), Davis et al. (1998), Vasfaee et al. (1998), Kim et al. (1999), Hoge et al. (1999), Mintun et al. (2002), Uludag et al. (2004), Ances et al. (2008), Griffeth et al. (2011, 2015), Liang et al. (2013), Germuska et al. (2019), Koush et al. (2019), and Zhang et al. (2019). Rat data from Collins et al. (1987), Ackermann and Lear (1989), Madsen et al. (1999), Dienel et al. (2002), Dienel et al. (2007), Cruz et al. (2007). All data are provided in Supplementary Table 3.

That glucose use increases linearly with increased rates of blood flow but glucose oxidation increases below linearity is paradoxical because blood is considered the sole source of both oxygen and glucose (as well as other nutrients) to the vertebrate brain. In the prevalent demand-based framework, if rCBF increased because of increased neuronal demand for oxygen, then one would expect to see the amount of blood oxygen extracted by the tissue (the oxygen extraction fraction, OEF) increase, as happens in skeletal muscle during exercise increasing to between 90 and 95% (Jie et al., 2014; Skattebo et al., 2020), or stay the same, as happens in the heart at approximately 70% (Lubberink et al., 2011; Lu et al., 2021). In contrast, during increased brain activity due to sensory stimulation, the fraction of blood oxygen extracted *falls*, from approximately 40% to below 30% [see Figure 1B; Buxton, 2010; Leithner and Royl, 2014].

**FIGURE 2:**
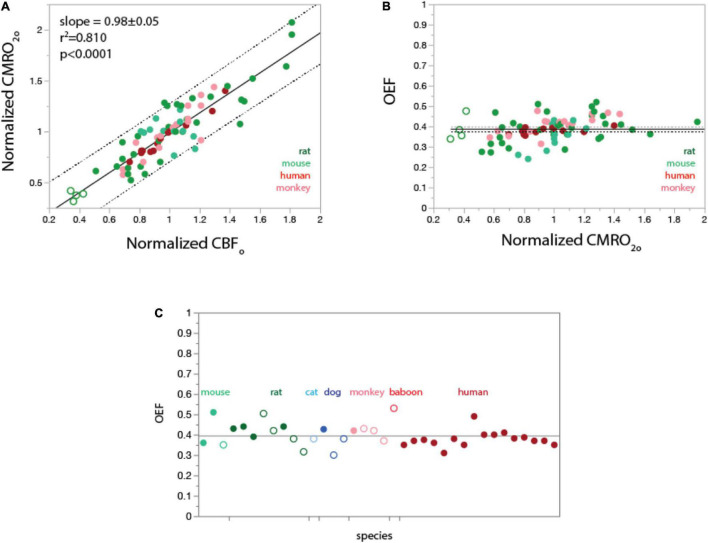
In the undisturbed awake state, rCMRO_2_ varies linearly with rCBF both within and across species **(A)**; as a consequence, regional OEF_o_ is constant at about 0.4 through different values of rCMRO_2_ **(B)** and across mammalian species **(C)**. **(A,B)** data from Frietch et al., 2000 (rat), Raichle et al., 2001 (human), Noda et al., 2002 (rhesus monkey), and Zhu et al., 2013 (mouse). Filled symbols indicate gray matter sites; unfilled symbols indicate white matter. Plotting of values normalized to the average for each species shows that regional CMRO_2_ varies linearly (fitted curve, linear function) with regional CBF both within the brain of each species and across species. **(B)**, Values of OEF_o_ calculated from data in **(A)** as the ratio of regional capillary oxygen diffusivity (rCMRO_2_) to regional CBF in the undisturbed awake state (rCBF_o_), given a constant PO_2_^A^. Values of OEF_o_ are constant around 0.4 within and across species, as predicted from the linear universal relationship between rCMRO_2_ and rCBF_o_ in **(A)**. Grand mean ± SD: 0.38 ± 0.06 (plotted). Species means ± SD: rat, 0.39 ± 0.06; mouse, 0.35 ± 0.06; rhesus monkey, 0.41 ± 0.05; human, 0.38 ± 0.02. **(C)**, Values of OEF_o_ reported in the literature for a total of seven mammalian species are also centered around a grand mean of 0.40 ± 0.05 (plotted). Filled symbols indicate awake animals; unfilled symbols indicate anesthesia. Data in C are from Kety (1956); Cohen et al. (1967); Vernheit et al. (1978); Laux and Raichle (1978); Raichle et al. (2001); Oshima et al. (2002); Kaisti et al. (2003); He et al. (2008); Ibaraki et al. (2008); Jain et al. (2010); Qin et al. (2011); Lu et al. (2012); Xu et al. (2012) (human); Hyder et al. (2016); Lee et al. (2017); Laux and Raichle (1978); Altman et al. (1991) (rhesus); Young et al. (1996), Noda et al. (2002) (baboon); Chen et al. (1984); Chang et al. (2016) (dog); Hossman et al. (1976) (cat); Johansson and Siesjo (1975); Nordstrom and Rehncrona (1977); Madsen et al. (1998); Linde et al. (1999); Frietch et al. (2000); Schmallbruch et al. (2001) (rat); He et al. (2008); Niwa et al. (2002); Zhu et al. (2013); Li et al. (2019) (mouse). All values are provided in Supplementary Table 1.

**FIGURE 3:**
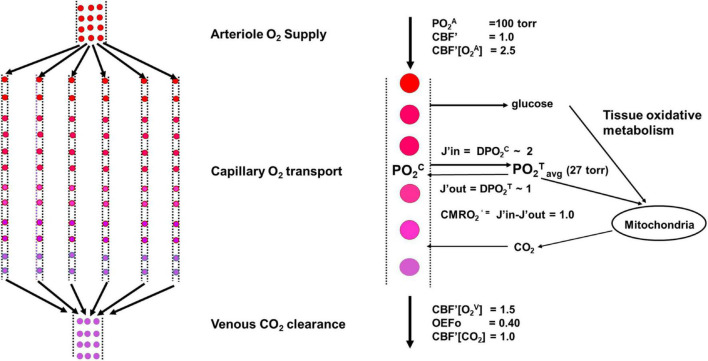
Schematic of the model in the undisturbed awake state.

**FIGURE 4:**
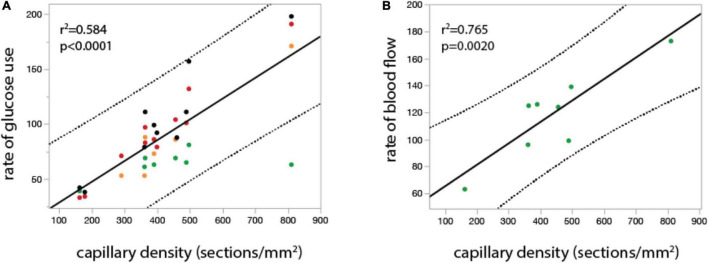
In the undisturbed waking state, local rates of glucose use and blood flow vary linearly across rat brain sites with local capillary density. Graphs show local rate of glucose use (**A**, in μmol.100mg^–1^.min^–1^) and local rate of blood flow (**B**, in ml. 100mg^–1^.min^–1^) plotted as a function of local capillary density (measured as number of capillary sections per mm2) in different structures in the rat brain in the undisturbed waking state. Both relationships are well fit by linear functions (r^2^ and *p*-value shown). Data from McCulloch et al. (1982), Nakai et al. (1990), Kuschinsky and Paulson (1992), and Levant and Pazdernik (2004), provided in Supplementary Table 2.

The prevailing demand-based framework of brain metabolism considers that a brain uses as much energy as its neurons *need*, which may change dynamically from one moment to the next, according to use, and may lead to ontogenetic modifications in the anatomy of the capillary bed that defines steady-state blood supply to the brain to match the different needs of different brain structures and species. In the context of evolutionary arguments, the demand-driven view has led to a large majority of papers proposing evolutionary selection of a balance between, on the one side, the advantage of more signaling, versus, on the other side, the disadvantage of the proportional increase in energy consumption. In these scenarios, what is referred to as “supply limitation” is avoided by physiological adaptations that ensure that demand is never limited, with the apparent excess supply during signaling, with a 2-3 fold excess of oxygen delivery by blood flow over consumption under all conditions, seen as a safety mechanism to ensure adequate oxygen delivery (Leithner and Royl, 2014).

Importantly, the demand-based framework assumes that oxygen and glucose supply to the adult brain through blood flow is plentiful, or at least not limiting, with ample room for accommodating changing demands. While the expectation that neurons have variable energy requirements that are dynamically, ontogenetically, and phylogenetically met by the blood supply on an as-needed basis makes intuitive sense in an ample supply scenario and appears to explain much of the experimental data, it is not necessarily correct. According to network transport theory (Banavar et al., 1999), metabolic rate, or the rate of energy use per unit time, should be understood first as a *consequence* of anatomically constrained energy supply, or availability, and how that in turn depends on the size of the body in question; only then should the balance between supply and demand be considered. In this case, the rate of energy use per neuron should be initially constrained by the relationship between local neuronal density (since neurons are the energy sink) and capillary density (since capillary walls are the energy source). If the limit imposed by supply is only reached during non-physiological activity such as seizure, then changes in neuronal activity may well constitute changes in “demand” (but see below), met by dynamic changes of the system whose balance remains to be understood. But if the anatomy of the capillary network imposes a limit to the physiology of the neurovascular system, as we propose here, then brain metabolism should be seen as a zero-sum game in which the activity of neurons in different brain regions remains balanced as a consequence of the limited, and limiting, oxygen and glucose supply, generating what appears as active “competition” but is simply the passive outcome of the near-maximal use of resources already at “rest.” Key to the zero-sum notion of whole-brain metabolism is that in physiological conditions, including mental activity and physical exercise, the rate of blood flow to the brain through the internal carotid artery is maintained steady, with the external carotid artery accommodating any increased flow rates from the heart (Sato et al., 2011; Smith and Ainslie, 2017).

We describe below how some of the apparent paradoxes in brain metabolism, such as the faster increase in rates of blood flow over glucose oxidation, are generated by the liberal use of terms that imply a demand-driven system. Here we take great care to avoid terms like “cost” and “demand” that imply neuronal agency, and use solely “energy supply” and “energy use” to analyze maximum and average energy consumption from the simple standpoint of delivery of substrate to the brain via blood flow, and then from the capillary to the tissue via a permeability limited concentration gradient.

We then propose an alternate view in which the relationships between brain energetics and brain function are fundamentally shaped by the physics of the relationship between capillary oxygen delivery capacity and CBF, given the blood pressure maintained across the brain. We start by describing how the wide acceptance of the view that the vascular system of the brain adapts to independently determined energetic needs is partially the result of the oft used terms “resting awake” versus “activation,” with the connotations of low versus high energy “demands.” We then perform a meta-analysis of studies from human and rat cerebral cortex over a wide range of metabolic activity (sleep to sensory stimulation and seizure) and find that the entire rCMRO_2_ to rCBF curve can be explained by limitations on capillary oxygen transfer and by changes in blood flow rates that maintain tissue pO_2_ and pCO_2_ homeostasis. We also show that these relationships are remarkably similar between different mammalian species and brain regions. These findings have implications for understanding fundamental constraints on brain function, estimating metabolic rates from species for which imaging is not available, and investigating the loss of brain function due to vascular dysfunction with aging.

### The Problem With the Terms “Demands,” “Requirements,” and “Resting”

Whether used as convenient shorthand or to convey a true sense of necessity, current understanding of brain metabolism has typically been framed in terms of neuronal “demands.” A key finding often cited to support this view was the seminal study of Attwell and Laughlin (2001), who showed that an energy budget built up from estimates of single neuron signaling energetics could explain *in vivo* findings of linear relationships between cerebral cortex neuronal oxidative glucose metabolism with glutamate neurotransmitter release (Sibson et al., 1998; reviewed in Rothman et al., 2019) as well as average neuronal firing rates (Hyder et al., 2013a). The expectation of a flexible supply adapting to a dynamically changing energy demand by neurons underlies the fundamental concept that blood flow can be used to track neuronal activity, critical for interpreting blood oxygen level dependent (BOLD) functional magnetic resonance imaging (fMRI) (Ogawa et al., 1990, 1993; Kwong et al., 1992).

We believe that the expression “neuronal demand” and its intuitive appeal have contributed to supply limitations on brain function by the neurovascular system remaining underexplored. An additional complication is that the concept of neuronal “demands” for energy implies agency, as if neurons knew what must be done and had any understanding of how to get there. A better alternative is to conceptualize “demand” as the sink end of a gradient (of concentration of oxygen or glucose, in this case), and “supply” as the gradient source. In this sense, then neuronal activity itself, whether spontaneous (ongoing) or under stimulation, is the energy sink, and will happen provided that there is a source of sufficient supply – and so “demand” becomes an unnecessary term. We thus propose that the expression “energy usage” is preferable because of its lack of teleological connotations.

A similar problem has arisen due to the use of the term “resting awake” state which derived from it being a control condition in human functional imaging studies in which the subjects received sensory stimulation or were asked to perform a mental task (Raichle et al., 2001). In the original interpretations of functional imaging studies, based on cognitive psychology theory, the “resting awake state” was expected to be a state of very low neuronal activity (Shulman and Rothman, 1998). As a consequence, findings from allometric studies regarding relationships between brain mass or neuronal number and the rate of energy metabolism have been considered of minor relevance to understanding functional brain metabolism. However, based on a range of experimental findings (Raichle et al., 2001; Raichle, 2006; Hyder et al., 2013b), this original concept of a resting brain has been replaced with the concept of a dynamic functional state with very high neuronal activity, even in the absence of structured stimulation, and metaphorically described as the brain’s “dark matter” (Raichle, 2006). Sensory, cognitive, and other inputs which were originally viewed as driving most of neuronal signaling in the brain are now largely seen as perturbations imposed upon an ongoing, undisturbed but active state (Raichle, 2006). To avoid the connotation of “rest” as a state of very little metabolic and neuronal activity, we use “undisturbed awake state” to refer to the quiet, awake state, under no particularly structured stimulation (when a subject is asked to perform mental processes or is given sensory stimuli they are directed to attend to).

### The Constancy of OEF Between Brain Regions and Species, Independent of Brain Mass, Supports a Common Relationship Between Oxygen Supply and Energy Consumption

Studies in the undisturbed awake state have shown a tight, linear correlation between energy supply (as measured through local rate of cerebral blood flow, rCBF) and energy use (measured as local rate of oxygen use, rCMRO_2_). In the rat brain, for instance, the inferior colliculus, the structure of highest glucose consumption and oxygen metabolism in the undisturbed awake state, also has the highest blood flow rate, and the white matter, the lowest rate of glucose use and lowest blood flow (Kuschinsky et al., 1981; McCulloch et al., 1982). It is tempting to conclude from this correlation alone that, as for the whole body (Banavar et al., 2002), brain metabolic rates are also largely determined by local rates of energy supply, that is, of both oxygen and glucose delivered together by rCBF. However, this conclusion appears in contradiction to the finding that over half of the oxygen delivered to the brain goes unused, as described by the oxygen extraction fraction (OEF). The OEF is experimentally defined as the ratio between regional measured rates of oxygen consumption and oxygen delivery (rCMRO_2_/([O_2_^A^]*rCBF)), where [O_2_^A^] is the arterial concentration of oxygen. The first measurements of OEF in human brain, obtained by arteriovenous difference studies, reported that the whole brain OEF is usually on the order of 0.40 (Kety, 1956; Cohen et al., 1967), which implies 60% of the oxygen delivered is not used, seemingly consistent with the view that oxygen supply from blood flow does not limit usage.

If oxygen supply from rCBF is not limiting, then there is no reason to expect a constant value of OEF in the undisturbed awake state, which we refer to as OEF_o_, across brain sites and species. Therefore, it was a surprise when Raichle et al. (2001), using positron emission tomography (PET), reported that the OEF_o_ of the undisturbed awake human brain is regionally highly uniform with a value of approximately 0.4. Similar results have been found in subsequent PET and MRI studies (Hyder et al., 2016). We therefore decided to test whether similarly uniform regional and average OEF_o_ values occur in other species.

We performed a meta-analysis of published studies in several species to see if there was a similar uniformity of OEF_o_ across brain sites in human and other species. All data and their sources are available in the Supplementary Information. Figure 2A shows a linear relationship in several different species between rCBF and rCMRO_2_. As a consequence, we find that OEF_o_, calculated as indicated above, is nearly constant at around 0.4 across various structures in human, rat, mouse, and rhesus brains (Figure 2B).

In order to obtain an estimate of the range of OEF_o_ as well as assess if there is any dependence on brain mass, we performed a meta- analysis on papers that have published whole cerebrum measurements of OEF_o_ in different species. A caveat to this analysis is the significant methodological variation in measurements of OEF, which can be influenced by anesthetics that change the coupling between blood flow and oxygen consumption (Franceschini et al., 2010). To try to account for these variations, we limited the data to awake animals and humans where possible, and in cases where the majority of data is from anesthetized animals, we only used data where anesthetics were believed to maintain closer to normal coupling (Kaisti et al., 2003). Figure 2 shows that when data from humans are limited to results from healthy adult non-elderly awake humans, the measured values of OEF_o_ are clustered around a mean of 0.38 ± 0.02, which we believe to provide an estimate of what the group measurement variation is due to differences in method. Figure 2C also shows OEF_o_ values from 6 other species with the filled circles being measurements obtained in awake animals and open circles anesthetized animals. It is seen that even with anesthetized animals included, the results cluster around an OEF_o_ of 0.40 ± 0.05 regardless of brain masses varying nearly 1,000-fold from mouse to human.

In support of the results of our meta-analysis, a recent ultra-high resolution optical mapping study that measured OEF_o_ in the awake mouse brain found values of OEF_o_ at the capillary level are tightly clustered around an average value of ∼ 0.4, with somewhat more homogeneity in deeper layers (Li et al., 2019). Therefore, the average OEF_o_ value of 0.4 we find in the undisturbed awake state reflects the majority of capillaries.

The constancy of the oxygen extraction fraction in the undisturbed awake state leads to a simple relationship between rCMRO_2o_, rCBF_o_, and OEF_o_:


(1) rCMRO2o=OEFo*rCBFo∼0.4*rCBFo

If rCBF_o_ and OEF_o_ were independently regulated, then in principle, the rate of oxygen delivery to brain tissue could be arbitrarily increased via rCBF_o_. However, the regulation of OEF, rCBF, and rCMRO_2_ are not independent but instead are interrelated in a way that maintains constant tissue average pO_2_ values (see below), as well as the products of oxidative metabolism such as PCO_2_, and pH (Buxton, 2021; Rothman et al., in preparation). Along with whole-body constraints on rCBF, these homeostatic constraints ultimately determine the maximum and average value of rCMRO_2_.

### The Ratio of Capillary Oxygen Delivery to Blood Flow Is Constant in the Undisturbed Awake State

Arguments for demand-controlled brain neuroenergetics often cite the increase in the rCBF to rCMRO_2_ ratio during structured stimulation, and the resulting drop in the OEF, as evidence of no supply limitation to brain oxygenation (Leithner and Royl, 2014). The reasoning here is that if O_2_ supply were limiting, then increasing the rate of blood flow should also increase the rate of O_2_ use. However, this view does not fully take into account the difference between delivery of O_2_ to the brain from the heart versus delivery of O_2_ to tissue via the capillary bed. A review of the average partial pressure of O_2_ in brain tissue, pO_2_^T^, shown in Table 1, demonstrates that pO_2_^T^ measured under awake conditions is close to the p50 of capillary hemoglobin, ranging in human cerebrum between 25 and 27 mmHg (Balcerek et al., 2020). Values in rats and mice are similar (Table 1). These values are approximately 10 torr above the threshold of approximately 12-15 Torr where pO_2_^T^ becomes potentially limiting for mitochondrial respiration (Buxton, 2010).

**TABLE 1:** Similarity of PO_2_^T^_avg_ across species.

Species	PO_2_^T^_avg_	Refs.
mouse	35.0	Moeini et al., 2018
	33.0	Moeini et al., 2020
	25.0	Lyons et al., 2016
	22.0	Gould et al., 2017
Average ± SD	28.8 ± 6.2	
rat	37.0	Almendros et al., 2011
	26.7	Dunn et al., 2011
	29.0	Dunn et al., 1999
	34.0	Liu et al., 1995
	25.0	Ortiz-Prado et al., 2010
Average ± SD	30.3 ± 5.0	
human	23.1	Pennings et al., 2008
	25.5	Longhi et al., 2007
	26.9	Narotam et al., 2009
	29.0	Spiotta et al., 2010
	33.0	Oddo et al., 2014
Average ± SD	27.5 ± 3.7	

*Average values of PO_2_^T^ (in torr) measured in the undisturbed, awake cerebral cortex of humans, rats, and mice. Although the available data is limited, it supports the conclusion that the average PO_2_^T^ value is species independent, despite a 3 to 4-fold range of variation in CMRO_2_.*

Importantly, pO_2_^T^ does not increase by more than a few percent with sensory stimulation and the ensuing variations in rCBF. Studies in mice using optical imaging have found that under conditions of intense sensory stimulation, there is an increase in PO_2_^T^ of only approximately 5% (Moeini et al., 2019). Thus, pO_2_^T^ is approximately constant independently of brain region, species, and metabolic rate. While the finding of a stable, low, but finite, PO_2_^T^ does not distinguish between demand-based and supply-limited scenarios of brain metabolism, it has been used to develop models based on oxygen delivery from capillary to tissue, which assume that the majority of oxygen exchange between blood and brain tissue occurs through the capillaries, despite some transfer from arterioles and venules (Jesperson and Ostergaard, 2012). Such models (Figure 3) have accurately predicted the relationship between rCMRO_2_ and rCBF in human cerebral cortex under different brain states (Hyder et al., 1998; Gjedde, 2005; Buxton, 2010; Leithner and Royl, 2014).

The figure shows a schematic of the model used under undisturbed awake conditions. A Krogh Erlang model was used in which the neurovascular unit was modeled as containing Nc parallel capillaries of length Lc (left). An expansion for a single capillary including the fluxes of oxygen into and out of the tissue is shown to the right. We describe the neurovascular system as being composed of four components: arteriole O_2_ supply by CBF; capillary O_2_ transport determined by effective oxygen diffusivity, proportional to the capillary length density and the capillary to tissue PO_2_ gradient; tissue oxidative metabolism of glucose; and venous clearance of products of respiration such as CO_2._ As shown in the main text, the regulation of CBF and capillary oxygen diffusivity (D) maintains a near constant value of PO_2_^T^_avg_ across the full range of CMRO_2_’ needed to support neural activity.

In these alternative models that we expand upon, the entire rCBF versus rCMRO_2_ curve derives from integrated regulation of the entire neurovascular system, rather than by excess oxygen delivery that is uncoupled through yet-undefined mechanisms. Put succinctly, these models consider that the non-linear increase in rCBF versus rCMRO_2_ is due to the progressive reduction in the efficiency of O_2_ transfer at higher flow rates as a consequence of constant capillary oxygen diffusivity. We extend these models here, focusing solely on the capillary part of the vascular distribution network in the brain, to show that, in combination with the finding in our meta-analyses of uniform values of OEF_o_ (Figure 2) and PO_2_^T^ (Table 1) across a range of species, these models of oxygen delivery predict that there is a constant species- and region-independent ratio between capillary oxygen transfer to brain tissue and blood flow to the same tissue volume in the undisturbed awake state.

The key regulatory components in our model are detailed in the Supplementary Information, and they interact as follows:

#### Cap of Maximal Capillary Oxygen Tension

The maximum capillary oxygen tension is capped at the input arterial oxygen tension (PO_2_^C^_max_ = PO_2_^A^). Once this value is reached, further increases in rCBF are ineffective at increasing the capillary-to-tissue O_2_ gradient, therefore placing a cap on rates of oxygen delivery and causing an apparent loss of efficiency of O_2_ transfer at higher values of rCBF.

#### Arteriole Regulation of Oxygen Supply by rCBF

The intrinsic limit on brain oxygen metabolism is the rate of delivery of oxygen by rCBF, at a rate of rCBF*[O_2_^A^], where [O_2_^A^] is the arterial concentration of oxygen, primarily bound to hemoglobin.

#### Capillary Regulation of Unidirectional O_2_ (and CO_2_) Transport

In capillary-based models of oxygen transport, based on the original work of Krogh in muscle (Krogh, 1919; Gjedde, 2005), the rate of net capillary oxygen transport from a capillary to the surrounding tissue (J) is given by the gradient between the average capillary and tissue oxygen tension, (PO_2_^C^_avg_ – PO_2_^T^_avg_), multiplied by a constant referred to as the effective capillary oxygen diffusivity per volume (D):


(2) J=D*(PO2Cavg-PO2Tavg)

Studies in the brain and other organs have shown that the effective capillary oxygen diffusivity D is independent of capillary radius and directly related to the capillary length density (D_C_), which is defined as the number of capillaries (N_C_) per volume of tissue (V) times the average capillary length (L_C_). Defining a dimensionless proportionality constant C_d_ and substituting D = D_C_ * C_d_ into Eq. [2] gives:


(2A) J=Cd*(NC*LC)/V)*(PO2Cavg- PO2Tavg)

#### Tissue Oxygen Metabolism

At steady-state, the net rate of capillary oxygen transfer (J) is equal to the rate of tissue oxygen metabolism via mitochondria (J = rCMRO_2_). In that case, the maximal rate of oxygen transfer from capillary to tissue, J_max_, is equal to the maximal rate of tissue oxygen metabolism, rCMRO_2max_:


(2B) Jmax=CMRO2max=CD*(Nc*Lc)/V)*(PO2A- PO2Tavg)

Eq. [1] can be solved numerically and analytically to determine the relationship between J and rCBF, provided D_C_, C_d_ and PO_2_^T^ are known, and assuming there is regular capillary geometry. The uniform values of OEF_o_ and PO_2_^T^ across species and brain regions shown in Figure 2 and Table 1 are empirical evidence of a fixed ratio between the rate between capillary oxygen transport, J, and rCBF.

To model this relationship, we used the reformulation of the Krogh model by Gjedde (2005) and Buxton (2010) in terms of OEF_o_ (for which there is substantial experimental data) and using the consideration that oxygen transfer across the capillary membrane is dependent on capillary length density. The term PO_2_^C^ (capillary pO_2_ average value) can be calculated from the O_2_ carrying kinetics of hemoglobin and the OEF, and then inserted into Eq. [2], yielding Eq. [3]:


(3) $$J={\text{C}}_{\text{d}}*\left({\text{N}}_{\text{c}}*{\text{L}}_{\text{c}}\right)/V)*[P50*{\left[\frac{2}{OEF}-1\right]}^{\left(\frac{1}{h}\right)}-PO2tavg]$$

In Eq. [3], P_50_ is the partial pressure of O_2_ at which half of hemoglobin oxygen carrying sites are occupied, h is the Hill coefficient, and D_O_ is the O_2_ diffusivity constant (in per volume units) in the undisturbed awake state. The values we use here for h and P50 are 2.8 and 50%, taken from Gjedde (2005) and Buxton (2010).

We used Eq. [3] to examine the ratio between capillary oxygen delivery to tissue (J_o_) and blood flow (rCBF_o_) in the undisturbed awake state across species and brain regions. After substituting OEF_o_ for measured OEF, J_o_ for J, and D_o_ for C_d_*(N_C_*L_C_)/V) into Eq. [3]; considering that J_o_ = OEF_o_*rCBF_o_*[O_2_^A^]; and rearranging, we derived the equation


(4) $$\frac{D_{o}}{rCBF_{o}}=\left(\frac{\left[O_{2}{}^{a}\right]}{P50}\right)*\frac{OEF_{o}}{{\left[\frac{2}{OEF_{o}}-1\right]}^{\frac{1}{h}}-\frac{PO_{2}{{}^{T}}_{avg}}{P50}}$$

where [O_2_^A^] is the oxygen concentration in the arterioles, which is proportional to the hematocrit and the arteriole partial pressure of oxygen (P_O2_^A^). Although there is a dependence of hemoglobin P50 and [O_2_^A^] on body mass, it is shallow (Dawson, 2014), so that to a first approximation, Eq. [4] simplifies to Eq. [5]:


(5) $$\frac{D_{o}}{{\mathrm{rCBF}}_{o}}=K1*\frac{{\mathrm{OEF}}_{o}}{{\left[\frac{2}{{\mathrm{OEF}}_{o}}-1\right]}^{\frac{1}{h}}-K2}$$

where K_1_ and K_2_ are constants. Thus, Eq. [5] shows that the value of OEF_o_ is predicted to depend only on the undisturbed awake state ratio of D_o_/rCBF_o_. As shown in Figure 2, our meta-analysis finding of only very small variations in OEF_o_ around an average of approximately 0.4 across brain regions and species implies that the D_o_/rCBF_o_ ratio also varies little, and is nearly constant. Mathematically, a constant D_o_/rCBF_o_ ratio requires that rCBF_o_ be strictly proportional to D_o_: that is, that the rate of blood flow in the undisturbed state vary across sites and species as a uniform, universal function of oxygen diffusivity, which in turn should be proportional to capillary length density (Krogh, 1919; Gjedde, 2005).

### Anatomical Evidence for the Relationship Between Do’ and Capillary Density

Under conditions of PO_2_^T^ homeostasis, then, the constant OEF_o_ of ca. 0.4 depends on capillary length density, or surface area, both in the undisturbed awake state and during higher levels of brain activity, assuming that oxygen transfer is primarily determined by capillary length density (Jesperson and Ostergaard, 2012). Recent studies have suggested that in mice, a significant fraction of oxygen delivery may also come from arterioles (Gould et al., 2017). However, in principle, given the similarity of the capillary bed geometry between species (Smith et al., 2019), Eq [5] will still hold with an effective oxygen diffusivity constant that includes the arteriolar contribution. Regardless, for now we focus on what is known about the relationship between capillary length density and the total rate of oxygen transfer, and how that relationship is consistent with our conclusion of a constant OEF_o_ and D_o_/CBF_o_ ratio.

Consistent with our proposition that rCBF_o_ is determined by the physical properties of the local capillary bed are the results of empirical studies that measured capillary density and rCBF_o_ and observed, as predicted, that rCBF_o_ (and also rCMR_glu_) in the undisturbed awake state is strongly linearly correlated with local capillary density (Klein et al., 1986; Borowsky and Collins, 1989). Figure 4 shows experimental results of a linear relationship between regional cerebral metabolic rate of glucose consumption (rCMR_glc_) or blood flow and local capillary density (see also Ventura-Antunes et al., 2022), as predicted from the meta-analysis in Figure 2 and Eq. [4]. In the undisturbed awake rat, approximately 95% of the glucose in the cortex is oxidized, which makes rCMR_glc_ an effective surrogate for rCMRO_2_ (Madsen et al., 1998). Therefore, the linear rCMR_glc_ x capillary density and rCBF x capillary density functions shown in Figure 4 are exactly what would be expected if J (and therefore CMRO_2_) was determined by local capillary length density: the higher the local capillary length density, the higher the rate of oxygen delivery to the tissue in the undisturbed waking state.

The key to our interpretation that local capillary surface area limits oxygen delivery to brain tissue, and therefore rCMRO_2_, in the undisturbed awake state is the extra constraint that tissue oxygen tension is kept approximately constant at a finite value (Figure 4), a constraint that was missed in previous comparisons of oxygen delivery across species (Karbowski, 2011). If PO_2_^T^_avg_ were not constant, variable rates of oxygen delivery at a given rCBF would be possible in a given capillary bed, a scenario that may occur under pathological conditions.

### The Problem Under Stimulation: Blood Supply Is Essential for Brain Function – But Is It Easily Changeable, Overly Abundant, Fixed and Limiting, or Just Right?

The second major issue we address from the standpoint of supply limitation is vascular uncoupling. Structured stimulation increases neuronal signaling rates which, through a variety of potential mechanisms, leads to increases in the diameter of the supplying blood vessels (arterioles and larger; Iadecola, 2017) that increase local CBF in the brain. In the undisturbed awake state, the relationship between the rate of net oxygen transfer via the capillary-tissue oxygen gradient (which is equal to rCMRO_2_ at steady state) and the rate of total oxygen supply via rCBF is linear, with a near constant slope of 0.40 that amounts to OEF_o_, as shown in Figure 2. This condition is often referred to as the neurovascular “coupled” state. In contrast, during structured sensory and other forms of stimulation that increase brain neuronal signaling, and as a consequence rCMRO_2_, there is a disproportionate increase in rCBF versus rCMRO_2_, with a drop in OEF, which has been referred to as neurovascular “uncoupling” and has been extensively documented (Buxton, 2010; Leithner and Royl, 2014). Importantly, variations in local rates of oxygen use are not due to differential coupling between rates of oxygen and glucose use across sites. Under non-stimulated conditions, approximately 90% - 95% of the glucose used is oxidized, producing approximately 27 molecules of ATP per glucose (Hyder et al., 2016; Blazey et al., 2018). However, during structured stimulation only approximately ½ of the increase in rCMR_glc_ is oxidized, which reduces the incremental ATP production to 16 per glucose (Hyder et al., 2016).

Figure 1 shows a plot of reported values from a meta-analysis we performed of the fractional increases in ΔrCBF and ΔrCMRO_2_ (ΔrCBF/rCBF_o_, ΔrCMRO_2_/rCMRO_2o_) from activated regions in the awake rat and human cerebral cortex during sustained sensory stimulation (visual in humans). Also plotted are theoretical curves for different slopes ranging from 1, anticipated if the linear coupling was maintained under stimulation, through 4, which is the maximum reported (reviewed in Buxton, 2010 and updated here). The best fit slope is 2.4 ± 0.1 for the human and rat data combined, implying an increase in total oxygen delivery far in excess of the increase in use under stimulated conditions. The resulting drop in OEF as ΔrCMRO_2_/rCMRO_2o_ increases is shown in Figure 1B.

Traditionally, neurovascular uncoupling has been proposed to indicate that oxygen delivery to the brain via the cardiovascular system is ample and thus rarely rate-limiting for metabolism. The “extra” vascular delivery of O_2_ has been further interpreted to provide an evolutionarily advantageous buffering capacity that provides a safety factor in case even more energy is needed for neuronal activity (reviewed in Leithner and Royl, 2014). With an almost 3-fold safety factor at maximum rCMRO_2_, supply might be construed to be never limiting. However, an alternate viewpoint is that while supply to the brain from the cardiovascular system is never limiting, rCMRO_2_ can never increase as much as rCBF if there are internal limitations to oxygen delivery in the system. We argue next for the latter scenario.

### Capillary Supply-Limited O_2_ Delivery Predicts the Relationship Between rCBF and rCMRO_2_ in the Undisturbed Awake State and During Structured Stimulation: Evidence From Studies in Human and Rat Cerebral Cortex

According to the concept of vascular uncoupling, any supply limitations that exist in the undisturbed awake state should not apply to stimulated conditions where OEF drops due to a relatively greater increase in rCBF than in rCMRO_2_ (see Figure 1). Under those circumstances, the brain in a state of supposed neurovascular uncoupling is, if anything, hyperoxygenated (Buxton, 2010; Leithner and Royl, 2014). Alternatively, it has been proposed (Buxton et al., 1998; Gjedde, 2005; Buxton, 2010) that the non-linearity of the rCBF x J curve during stimulation is not due to uncoupling, but rather reflects a compensatory increase in rCBF given a reduced efficiency of O_2_ transfer to brain tissue.

Here we extend this alternative proposition by hypothesizing, based on our meta-analysis and experimental findings (Ventura-Antunes and Herculano-Houzel, 2022; Ventura-Antunes et al., 2022), that the limitations to O_2_ transfer to brain tissue are the same across mammalian species, explained by whatever mechanisms set PO_2_^T^ homeostasis and capillary length density resulting in an OEF_o_ of ca. 0.4. Therefore, we predict that brain regions across all species share the same rCBF x rCMRO_2_ relationship during stimulation. To test this possibility, we normalized Eqs. [3] and [4] in terms of the ratio of rCBF and net oxygen delivery (J) relative to their values in the undisturbed awake state (J’ = J/J_o_, rCBF’ = rCBF/rCBF_o_), so that the measured rCBF x rCMRO_2_ values from different brain regions and species could be compared:


(6) $$rCBF^{\prime }=\left(J^{\prime }(\frac{OEF_{o}}{2})\right)(1+{\left[J^{\prime }*\left(\frac{J_{o}}{D_{o}*P50}\right)+\frac{PO_{2}{{}^{T}}_{avg}}{P50}\right]}^{h}$$

We note that the non-normalized version of Eq. [6] has been derived in previous publications (Buxton et al., 1998; Gjedde, 2005; Buxton, 2010).

**FIGURE 5:**
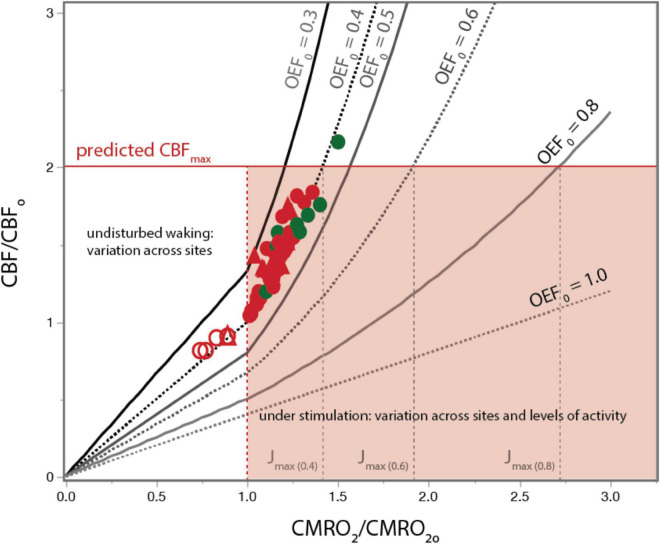
A constant ratio of D_O_/rCBF_O_ predicts the relationship between rCBF and rCMRO_2_ over the full range of cortical brain activity in humans and rats. Red (human) and green (rat) circles show measured values of rCBF normalized to average rCBF_o_ as a function of rCMRO_2_ normalized to average rCMRO_2o_ both in the undisturbed state (straight lines up to CMRO_2_/CMRO_2O_) and during photic stimulation (curved lines, rCMRO_2_/rCMRO_2O_ > 1.0). Empirical values across states are very well aligned with values predicted by our model using OEF_o_ = 0.4. Using a higher OEF_o_ = 0.5 already underestimates K_CBF_, and OEF_o_ = 0.3 overestimates it. Solid red line indicates the empirical maximal rCBF/rCBF_o_; dotted lines indicate the corresponding predicted values of J_max_ for each OEF_o_. Data provided in Supplementary Table 4.

The value of J_o_/D_o_*P50 was determined using Eq. [3] for different values of OEF_o_ ranging from 0.3 to 1.0 in the resting awake state. Unfortunately, there is limited data available on the rCBF versus rCMRO_2_ relationship in awake nonhuman animals. However, there are several published studies in awake rats undergoing sensory stimulation using autoradiography techniques from which this relationship can be determined, and many studies in human cerebral cortex. In Figure 5 we plotted these values, along with results of studies using calibrated fMRI and PET of human cerebral cortex during visual stimulation. We restricted the human studies to visual stimulation because the paradigms are relatively standard and reproducible and, most importantly, visual stimulation activates a large area of cortex which reduces the possibility of the measured values of the increase in rCBF and rCMRO_2_ being artificially low due to the imaging volumes also containing non-stimulated cortex.

As shown in Figure 5, there is excellent agreement between measured values of rCMRO_2_’ and rCBF’ obtained in rat (green) and human (red) cerebral cortex during sensory stimulation and seizure with the values predicted in Eq. [6] for an OEF_0_ of 0.40, despite the absolute rCBF and rCMRO_2_ values for the rat being almost 3 fold higher. The agreement between the measured rCBF’ x rCMRO_2_’ relationship and our model’s predictions for an OEF_0_ of 0.40 strongly supports our hypothesis that a constant value of D_o_/rCBF_o_ imposed by the physical properties of the capillary bed, given pO_2_^T^ homeostasis, determines the rCBF x rCMRO_2_ relationship across all brain states.

The calculations above are for values rCMRO_2_ above the undisturbed awake state, over which capillary oxygen diffusivity is close to constant due to lack of capillary recruitment. However, *below* the undisturbed awake state, in all brain regions there is a near linear decrease in D and rCBF with respect to the decrease in rCMRO_2_ (Hyder et al., 1998; Jesperson and Ostergaard, 2012). We incorporated this linear relationship in our model based on Hyder et al. (1998) by making D *below* the undisturbed awake state proportional to rCBF’ so that D = D_o_*rCBF’. As shown by the unfilled symbols in Figure 5, this modification accurately predicts the published human data for light deprivation and deep sleep.

**FIGURE 6:**
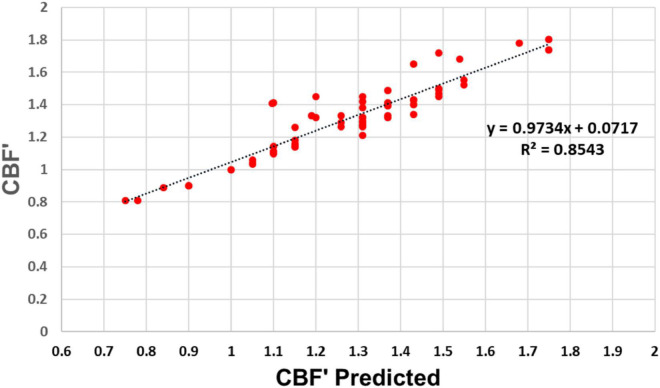
Experimental versus predicted rCBF′ across the full range of rCMRO_2_. Experimental and predicted values of rCBF′ according to our model are linearly related with a slope of 0.97 and r^2^ = 0.854, indicating that our model accounts for 85.4% of the variation in the experimental data.

To further assess how well experimental values agree with our model’s predictions, we plotted in Figure 6 the measured versus predicted values of rCBF/rCBF_o_ for each data point in Figure 5. Again, we find excellent agreement for the human data, with a slope of 0.97 and an r^2^ of 0.85 (Figure 6).

### A Fixed D_o_/rCBF_o_ Ratio Imposed by Capillary Density, PO_2_^T^ Homeostasis, and Physiological Limitations on rCBF Determine the Maximum Normophysiological Value of rCMRO_2_

Figure 5 shows experimentally and theoretically that the rCBF versus rCMRO_2_ relationship becomes increasingly non-linear at higher values of rCMRO_2_, resulting in a progressive OEF decrease. The non-linearity is due to the fixed value of D at near the undisturbed awake value D_o_, accompanied by homeostatic regulation of rCBF that maintains PO_2_^T^. As described in Eq. [2B], the capillary to tissue PO_2_ gradient approaches a maximum value with PO_2_^C^_avg_ = PO_2_^A^. At low values of OEF_o_, the undisturbed awake value of PO_2_^C^_avg_ starts closer to PO_2_^A^, leaving less room for an increase in the gradient. Thus, because increased rCBF cannot raise the value of capillary pO_2_ past arterial pO_2_, the oxygen gradient described in Eq [1] is capped at pO_2_^A^ regardless of further rCBF increases. Thus, arterial blood oxygenation imposes a practical limit to how much the net rate of capillary oxygen delivery to tissue (J) can be increased by increasing rCBF.

There is yet another limitation on maximal rates of tissue O_2_ delivery due to the blood supply to the brain. Other than during pathological hypertension, as occurs during seizure, maximum rCBF does not exceed 2 times the undisturbed awake value. This limit may reflect the relatively constant blood pressure drop in the brain across species (Karbowski, 2011). Assuming this generous upper limit of 2 x rCBF_o_, Figure 5 shows that the maximum sustainable increase in rCMRO_2_ during stimulation over the undisturbed awake state is only 1.4-fold, that is, at best 40% above undisturbed waking, even in conditions where rCBF is double the rCBF_o_. In the studies we reviewed, this value of J was only exceeded for the rat brain under conditions of chemically induced seizure (Ackermann and Lear, 1989).

**FIGURE 7:**
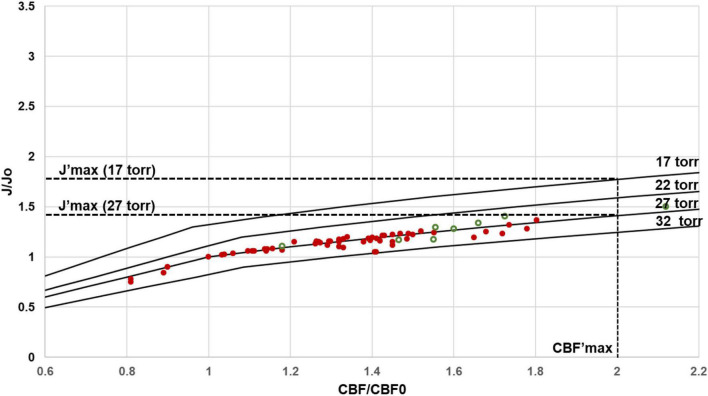
Predicted regional J/Jo (J′) versus regional CBF/CBF_o_ (CBF′) for different values of PO_2_^T^_avg_. At the measured value of OEF_o_ = 0.40, human (red filled circles) and rat (green open circles) data closely agree with the J′ versus rCBF′ curves calculated with an activity independent PO_2_^T^ of ∼ 27 torr. Note that even small variations in PO_2_^T^_avg_ would be detectable. Regional CMRO_2_ in principle can be increased by reducing PO_2_^T^_avg_. For the lowest value of PO_2_^T^_avg_ required to support mitochondrial function, approximately 17 torr, J′_max_ (at CBF′_max_ of 2.00) is 1.75, versus 1.42 at the physiological PO_2_^T^_avg_ value of 27 torr.

While capillary density and rCBF are the major determinants of the net rate of capillary oxygen transport to brain tissue, which equal rCMRO_2_, the partial O_2_ pressure in the tissue is also relevant. As shown in Eq. [2], O_2_ delivery could be increased if PO_2_^T^_avg_ could be reduced. Figure 7 shows the predicted J’ versus rCBF’ curves for a constant PO_2_^T^_avg_ of 27 torr, consistent with experimental measurements in the undisturbed wake state (Table 1), as well as other curves calculated for constant values of PO_2_^T^ ranging from 15 torr to 40 torr. As seen in Figure 7, *in vivo* results are highly consistent with a constant PO_2_^T^ average centered on 27 torr. It has been proposed that this finite value compensates for the further drop in tissue PO_2_^T^ from the outside of the capillaries to the mitochondrial membrane (Hudetz, 1999; Mintun et al., 2001; Gjedde, 2005; Buxton, 2010; Jesperson and Ostergaard, 2012; Leithner and Royl, 2014). However, it has been shown in hypoxia studies that brain oxidative metabolism can transiently be sustained at an average P_O2_^T^ of ∼15 torr (Mintun et al., 2001). Based on the calculations of Figure 7, a lower PO_2_^T^ of 15 torr at a CBF’ of 2.0 would only increase the maximum value of J’ from 1.40 to 1.75, still below 2.0.

### Why Is the Undisturbed OEF Only ∼ 0.4? Implications for Evolutionary Selection of Capillary Properties

Because evolutionary arguments often consider that biological features have been optimized through selection, we also explore in Figure 5 how the theoretical relationship between rCBF and rCMRO_2_ would differ if capillary oxygen diffusivity were decreased (resulting in lower OEF_o_) or increased (resulting in higher OEF_o_). If selection pressure acted to maximize energy supply to the brain, within the limits of the cardiovascular system, we would expect that capillary diffusivity would be maximized within anatomical constraints. With OEF_o_ = 0.3, larger increases in rCBF relative to rCBF_o_ would indeed be required to sustain a similar level of tissue oxygen delivery during structured stimulation, and the maximal increase of rCMRO_2_ over the undisturbed state would be of less than 25%. In contrast, with higher values of OEF_o_, and the associated D_o_, there is much more room for further increases in rCMRO_2_ before rCBF even approached its maximum, as shown in Figure 5. For example, at the physiological PO_2_^T^ of 27 torr, a higher OEF_0_ of 0.6 compared to the measured 0.4 would increase rCMRO_2max_ from 1.4 to 1.8-fold over rCMRO_2o_.

Although the argument has been made that higher OEF values could lead to hypoxic regions in the venous end of the capillary bed, this has been theoretically and experimentally shown to be unlikely to be limiting even for values of OEF as high as 0.8 (Hudetz, 1999; Mintun et al., 2001; Jesperson and Ostergaard, 2012; Leithner and Royl, 2014). In studies in humans undergoing visual stimulation during mild hypoxia, which increases OEF, there was no impact on rCMRO_2_ even with an increase of OEF to 0.8 (Mintun et al., 2001). These findings indicate that higher values of OEF_o_, which would be beneficial to rCMRO_2max_, are theoretically possible, in which case the relatively low OEF_0_ of 0.4 must reflect a fundamental biological constraint – such as one imposed by the capillary density of brain tissue. Higher values of OEF_o_ for a same rCBF value would require greater local capillary density, but because local capillary density and rCBF vary linearly across brain sites, any small increases in local capillary density would boost both rCBF and OEF_o_. While any increases in capillary density would reduce the brain volume available for neurons and glia, the volume fraction of brain tissue occupied by capillaries is very small, typically between 1 and 4% of the parenchyma (Ventura-Antunes and Herculano-Houzel, 2022; Ventura-Antunes et al., 2022), which further suggests that there is literally room for higher capillary densities in the brain.

The metabolic restriction imposed by the low capillary O_2_ diffusivity of the brain is highlighted by comparing it with muscle. Whole brain CBF in the undisturbed awake brain is as high as blood flow rates in skeletal muscle (SMBF) during aerobic exercise such as cycling, over 40 ml.min^–1^.100g^–1^, which are about 10-fold higher than SMBF at rest (Madsen et al., 1993; Jie et al., 2014). Unlike the brain, where OEF drops with increasing rCBF and hardly any effective capillary recruitment, the OEF of muscle increases from 70% at rest to 95% as capillaries are recruited and maximum oxygen consumption rate is reached. An OEF of approximately 70% has been reported for heart that is also maintained at high workload (Lubberink et al., 2011; Lu et al., 2021). The ability of exercising muscle and heart to sustain high OEF values supports our conclusion that limitations in capillary oxygen transfer capacity constrain rCMRO_2max_. Therefore, from a standpoint of blood flow and oxygen consumption, the undisturbed brain is more like the exercising muscle.

Given the finding that, across species with a substantial evolutionary separation and range of brain sizes, the physiological OEF_o_ is fairly low, at 0.4, and is so restrictive to oxygen supply to brain tissue suggests that O_2_ delivery is *not* optimized to support maximum brain energetic requirements, but rather *constrained* by other factors that determine capillary density in the tissue. What factors determine capillary density in the developing brain, and thus the relationship between OEF_o_ and CBF_o_ in the adult brain, is a topic for future investigation. It is possible that capillary density self-regulates in development in a manner that maintains pO_2_/pCO_2_ homeostasis (Buxton, 2021), which may require an OEF_o_ ∼0.4 for the optimum ratio (Rothman et al., in preparation). In addition, brain tissue volume is also a likely major determining factor of capillary density and CBF for purely physical reasons (Banavar et al., 1999). However, these factors are not independent since, as shown in Eq. [5], the maintainance of pO_2_^T^ homeostasis requires that capillary density and rCBF are mathematically interrelated. Surprisingly, local neuronal density does not seem to matter, for there is no consistent correlation between local capillary density and local neuronal density in rodent brains (Ventura-Antunes and Herculano-Houzel, 2022; Ventura-Antunes et al., 2022). The validity of this framework of restricted delivery of O_2_ depending on local capillary density opens a new horizon for extending comparative studies of capillary densities in brain tissue, including the possibility of inferring brain metabolic rates in species for which functional measurements are unlikely to become available, for example in fixed brain tissue of species that cannot be brought to the lab. We expect that these studies will greatly expand our understanding of the allometric scaling of brain energetics across species.

### The Uncoupling That Is not, and the Implications of Functioning Near Capacity

The continuity of the agreement between predicted and experimental values both in the undisturbed state and during structured stimulation shows that the apparent mismatch between changes in rates of rCBF and rCMRO_2_ that has been described in the literature as “neurovascular uncoupling” can be largely explained by a supply-limited framework of brain metabolism in which the anatomy of the capillary bed combined with a constant rate of capillary oxygen diffusivity under conditions of the maintenance of tissue O_2_ homeostasis imposes constraints to total oxygen delivery.

More specifically, our meta-analysis supports that constraints to total oxygen delivery (measured as the rate of whole brain CMRO_2_) are imposed by three main factors at play across species, across brain regions, and over time: (1) the capacity for delivering oxygen to the brain via the cardiovascular system, limited by the rate of blood flow at the internal carotid and vertebral arteries; (2) the capillary (length) density of the parenchyma, which restricts oxygen delivery from blood to brain tissue, and (3) continued maintenance of PO_2_^T^ (and potentially pCO_2_^T^ (Buxton, 2021), and pH) homeostasis. Previous meta-analyses of activation (e.g., Buxton, 2010) have focused largely on the changes in CBF and rCBF versus increases in CMRO_2_ and rCMRO_2_ (see Figure 3). However, Figure 1 shows that when results are plotted as normalized values of rCBF versus normalized values of rCMRO_2_, even with variations across labs and methods, the results are all consistent with the curve largely being determined by capillary effective oxygen diffusivity (which depends on capillary length density) and pO_2_^T^ homeostasis. Further, based upon the constancy of OEF_o_ in the undisturbed awake state across brain regions and mammalian species, we predict that the same rCBF x rCMRO_2_ curve applies throughout, and not as an “optimized” physiological feature, but one that follows naturally from what we propose to be mostly supply-limited brain metabolism, with capillary density as the key limiting factor to the rate of oxygen delivery to brain tissue across brain states.

We propose that it is in this context of supply-limited brain metabolism already in the undisturbed waking state that activity-dependent changes in blood flow occur, generating the decreased OEF and increased hemoglobin fractional oxygenation that constitute the BOLD signals that are monitored in fMRI. Importantly, the ability of our model to predict the rCBF x rCMRO_2_ relationship across states indicates that there is no *qualitative* difference in the neurovascular support of brain metabolism between “rest” and “stimulated” states, that is, between undisturbed waking and during sensory stimulation. As previously concluded from the consideration of pO_2_^T^ homeostasis alone (Gjedde, 2005; Buxton, 2010), it is incorrect to invoke an “uncoupling mechanism” whereby rCBF is elevated in excess of oxygen metabolism during sensory stimulation. Much to the contrary, we show that the rate of oxygen metabolism remains coupled to the effective rate of oxygen delivery, which is limited by capillary diffusivity and therefore by local capillary densities, at all modeled states, including when rCBF becomes “disproportionately” more elevated than regional oxygen metabolism. Put another way, the constancy of the capillary diffusivity to oxygen delivery (via CBF) ratio, in combination with the constraint of approximate PO_2_^T^ homeostasis, locks in the rCBF x rCMRO_2_ relationship regardless of brain state. Thus, there is no uncoupling.

We believe that further developing our understanding of how the anatomy of the blood supply network limits brain energy metabolism can potentially have important diagnostic potential for differentiating deterioration in brain function due to oxygen supply from the blood versus disturbances of intrinsic metabolic capacity. Increasing evidence exists that in healthy aging, and even more so with pre-Alzheimer’s and other neurodegenerative conditions, there are substantial vascular changes which may reduce delivery capacity (Brown and Thore, 2011). Although beyond the scope of this paper, the simulations in Figures 5, 7 can be used to make predictions about at what level impairments in brain perfusion will impact brain function. Based on Figure 7, given a constant ratio of D_o_/rCBF_o_ (that is, constant ratio of capillary density to rCBF), and at the cost of a decrease in PO_2_^T^ to 15 torr, the maximum value of rCMRO_2_ with J’ of 1.4 could be sustained with a 62.5% lower rCBF’_max_ of 1.25 instead of 2.0. However, studies on mild hypoxia have found that a PO_2_^T^ of 15 torr would impair aspects of cognition such as speed of task completion (Nakata et al., 2017). We predict that even a 25% reduction in capillary oxygen diffusivity, which would reduce OEF_o_ to 0.3, would severely limit rCMRO_2max_ and brain function (Figure 5). This prediction is consistent with reports of impaired performance and rCMRO_2_ responses to sensory stimulation and cognitive tasks in elderly subjects in fMRI studies (Hutchinson et al., 2013). Although preliminary, the calculations above demonstrate the diagnostic potential of models that include homeostatic and anatomical constraints on the neurovascular system to interpret the bases of altered rCMRO_2_ and rCBF in the elderly. We are currently working to test the diagnostic potential of our supply-limited model of brain metabolism.

Finally, we wish to speculate that a limitation to brain metabolism imposed by the capillary supply of blood and oxygen to the tissue already in the undisturbed state would also account for two main findings of neuroimaging and psychology: the trade-off across functional networks and the attentional limitation to a single focus. This limitation is all the more important since blood circulation is a closed system, where diverting more blood flow to one site necessarily occurs at the expense of decreased flow to all other sites. Similarly, blood circulation in the brain must also be treated as a closed system, given that under physiological conditions, the entire capillary bed is already perfused with blood even in the undisturbed awake state (Mintun et al., 2002), and the rate of arterial blood flow to the cerebrum is tightly controlled and remains steady even during extreme rates of blood flow through the heart, as in intense exercise (Sato et al., 2011).

In contrast to resting muscle, the brain in the undisturbed awake state already has a very high level of neuronal activity and consequent metabolic activity (Raichle et al., 2001; Raichle, 2006; Hyder et al., 2013b), which Raichle (2006) has referred to as the brain’s “dark energy”. To support this metabolic activity, CBF in the undisturbed awake state is already high, capable of undergoing a maximum regional increase of at most 2-fold under stimulation, with minimal reported whole brain increases under physiological conditions. Given the *whole brain* constancy of CBF even during intense stimulation (Mintun et al., 2002), increases in *local* CBF occur at the expense of decreases in CBF in other locations, as was shown with the observation of negative BOLD signals across large brain areas during tasks (Raichle et al., 2001). Sensory stimulation makes this trade-off particularly conspicuous, but the fact that local increases occur at the expense of decreases in brain metabolism elsewhere also became apparent in later studies of longitudinal variation in brain metabolism at “rest” (Biswall, 2012). Importantly, the locations involved in such trade-offs are not random; as confirmed by studies of functional connectivity, even in the undisturbed awake state, increases and decreases in local activity occur jointly across structures that participate in the main functional networks in the brain (Smith et al., 2013). It thus appears that what the human brain experiences as an alternation over time between a focus on orienting on the external world, or coordinated actions of the hands, or internal focus on the self, or planning future actions might be not an evolved or adaptive benefit from a computational standpoint, but rather a consequence of a fundamental limitation of brain metabolism imposed by the combination of intense neuronal activity already at “rest” in the face of a given, limiting O_2_ supply due to limitations on CBF and capillary density.

Similarly, we propose that our attentional limitation to one focus at a time (James, 1890) might result not from selection of attentional circuits that function this way over other possible configurations, but quite simply from the necessary consequence of diverting blood flow under supply-limiting conditions: we point out that, due to whole brain CBF being held constant, the brain circulation functions as a closed system under physiological conditions, and therefore any more blood supplied to a site of intense, focal increase in activity occurs at the expense of less blood supplied to all other sites. Indeed, a recent study using oxidated states of cytochrome oxidase as an indicator of brain metabolism during changing visual attention demonstrated such a compensatory mechanism (Bruckmaier et al., 2020). Also consistent with this proposal are visual studies in humans and non-human primates that have shown that attending to a region in the visual field enhances the neuronal response in the visual cortex to stimuli occurring at that region and suppresses the neuronal and vascular response to stimuli in other regions (Kastner and Ungerleider, 2000). The enhanced response requires attention as opposed to only awareness (Watanabe et al., 2011). In this scenario, we thus propose that the neural correlates of local and global mental processes are fundamentally limited regionally by maximum oxygen delivery and globally by the near constancy of whole-brain CBF under physiological conditions. Enhanced brain processing moves from functional network to functional network, from modality to modality, and from site to site within a modality, as each local winner metaphorically, and temporarily, takes all, or more, from a resource that is in limited supply.

## Data Availability Statement

The original contributions presented in the study are included in the article/Supplementary Material, further inquiries can be directed to the corresponding author/s.

## Author Contributions

SH-H and DR developed the theoretical framework and wrote the manuscript. DR compiled and analyzed the dataset. SH-H prepared figures and tables. Both authors contributed to the article and approved the submitted version.

## Conflict of Interest

The authors declare that the research was conducted in the absence of any commercial or financial relationships that could be construed as a potential conflict of interest.

## Publisher’s Note

All claims expressed in this article are solely those of the authors and do not necessarily represent those of their affiliated organizations, or those of the publisher, the editors and the reviewers. Any product that may be evaluated in this article, or claim that may be made by its manufacturer, is not guaranteed or endorsed by the publisher.
