# Supplementary material for: From a Demand-Based to a Supply-Limited Framework of Brain Metabolism
Source: Front Integr Neurosci. 2022 Apr 1;16:818685. doi: 10.3389/fnint.2022.818685 (PMC9012138; doi:10.3389/fnint.2022.818685)
Supplement: Supplementary file 1 [file Data_Sheet_1.pdf]

Supplementary Information

Parameters used in the model calculations

The following parameters and variables were used in the calculations of the simulated J x CBF curves. The parameters and variables were adopted from Gjedde (2005) and Buxton (2010). We note that although there are variations in the values of parameters between species, they will have a minor impact on the calculated curves.

J Net rate of oxygen delivery from capillaries to tissue, per volume and unit time.

J_o Net rate of oxygen delivery per volume and unit time in the undisturbed awake state.

$[O_2]^A$ Arterial oxygen concentration

D_o Average capillary O_2 diffusivity constant in the undisturbed awake state, in units of oxygen transfer rate per unit volume. Its value depends upon the total capillary length density (see main text) and the hematocrit.

OEF Oxygen extraction fraction between the arteriole input and venule output of a capillary bed.

OEF_o Oxygen extraction fraction in the undisturbed awake state

PO_2^T Average tissue partial O_2 pressure in torr (set to 27 torr).

PO_2^C Average capillary partial O_2 pressure in torr.

PO_2^A Arterial partial O_2 pressure in torr.

$P50^C$ Partial O_2 pressure at which hemoglobin is saturated at 50% in capillaries (set to 27 torr)

h^C Hill exponent in capillary blood (set to 2.8).

Additional Description of Meta-Analysis and Calculations

Measured values of CBF and $CMRO_2$ given and described in the tables below were used for all calculations along with the equations given in the text. Experimental descriptions are provided in the tables and in more detail in the original references. All values were normalized to the reported values in the undisturbed awake state. In the case of calibrated fMRI studies, the data in the publications were expressed as normalized values. For rat cerebral cortex activation studies, in several cases rCBF' was estimated from the fractional increase in cerebral glucose metabolism (rCMRglc'), which has been shown to be an accurate surrogate for rCBF' (Mintun et al., 2002).

Table S1. Data used in Figure 1, undisturbed awake or anesthetized state.

Species	Site	Normalized CBF_o	Normalized $CMRO_{2o}$	OEF	Ref
Human	A	1.37	1.40	0.404	Raichle et al., 2001
	B	0.735	0.70	0.360	Raichle et al., 2001
	C	0.813	0.81	0.395	Raichle et al., 2001
	D	1	1.00	0.391	Raichle et al., 2001
	E	0.991	0.99	0.392	Raichle et al., 2001
	F	0.874	0.80	0.361	Raichle et al., 2001
	G	0.893	0.81	0.355	Raichle et al., 2001
	H	0.813	0.79	0.377	Raichle et al., 2001
	I	0.94	0.93	0.388	Raichle et al., 2001
	J	1.284	1.20	0.373	Raichle et al., 2001
	K	0.92	0.88	0.372	Raichle et al., 2001
	L	1.11	1.06	0.378	Raichle et al., 2001
	M	0.821	0.81	0.389	Raichle et al., 2001
Rat	Cortex	0.808	0.656	0.318	Frietch et al., 2000
	Dentate nu.	1.477	1.311	0.347	Frietch et al., 2000
	Vestibular nu.	1.384	1.445	0.409	Frietch et al., 2000
	Cochlear nu.	1.496	1.296	0.340	Frietch et al., 2000
	Superior olive	1.775	1.639	0.362	Frietch et al., 2000
	Pontine grey	0.938	0.700	0.292	Frietch et al., 2000
	Lateral lemniscus	1.468	1.073	0.286	Frietch et al., 2000
	Inferior colliculus	1.812	2.071	0.448	Frietch et al., 2000
	Superior colliculus	1.087	0.998	0.360	Frietch et al., 2000

	Substantia nigra	0.929	0.909	0.383	Frietch et al., 2000
	Substantia nigra	0.836	0.581	0.272	Frietch et al., 2000
	Medial geniculate body	1.551	1.520	0.384	Frietch et al., 2000
	Lateral geniculate body	0.994	1.043	0.411	Frietch et al., 2000
	Mammillary body	1.180	1.088	0.361	Frietch et al., 2000
	Hypothalamus	0.725	0.581	0.314	Frietch et al., 2000
	Ventral thalamus	1.041	0.998	0.376	Frietch et al., 2000
	Lateral thalamus	1.115	1.118	0.393	Frietch et al., 2000
	Hippocampus CA1	0.688	0.894	0.510	Frietch et al., 2000
	CA2	0.650	0.656	0.395	Frietch et al., 2000
	CA3	0.762	0.775	0.399	Frietch et al., 2000
	CA4	0.790	0.954	0.473	Frietch et al., 2000
	Dentate gyrus	0.688	0.730	0.416	Frietch et al., 2000
	Amygdaloid complex	0.743	0.522	0.275	Frietch et al., 2000
	Globus pallidus	0.511	0.611	0.469	Frietch et al., 2000
	Caudate nucleus	0.966	1.282	0.520	Frietch et al., 2000
	Nucleus accumbens	0.994	1.028	0.405	Frietch et al., 2000
	Visual cortex	1.078	1.252	0.455	Frietch et al., 2000
	Auditory cortex	1.812	1.952	0.422	Frietch et al., 2000
	Parietal cortex	1.050	1.267	0.473	Frietch et al., 2000
	Sensory motor	1.273	1.341	0.413	Frietch et al., 2000
	Frontal cortex	1.152	1.326	0.451	Frietch et al., 2000
	Pyriform cortex	0.985	1.252	0.498	Frietch et al., 2000
	Lateral septal nuclei	0.725	0.641	0.347	Frietch et al., 2000
	Internal capsule	0.381	0.372	0.383	Frietch et al., 2000
	Corpus callosum	0.344	0.417	0.476	Frietch et al., 2000

	Genu of corpus callosum	0.362	0.313	0.339	Frietch et al., 2000
	Cerebellar white matter	0.427	0.387	0.355	Frietch et al., 2000
Mouse	1	0.83	0.989	0.41	Zhu et al., 2013
	2	1.09	1.005	0.32	Zhu et al., 2013
	3	0.81	1.001	0.43	Zhu et al., 2013
	4	0.87	1.017	0.41	Zhu et al., 2013
	5	1.03	1.001	0.34	Zhu et al., 2013
	6	0.96	1.005	0.36	Zhu et al., 2013
	7	1.17	0.829	0.24	Zhu et al., 2013
	8	0.91	1.128	0.43	Zhu et al., 2013
	9	1.07	1.214	0.39	Zhu et al., 2013
	10	1.07	1.095	0.36	Zhu et al., 2013
	11	1.16	0.952	0.28	Zhu et al., 2013
	12	1.03	0.763	0.26	Zhu et al., 2013
Rhesus monkey	Cerebellum	0.826	0.701	0.349	Noda et al., 2002
	Hippocampus	0.931	0.841	0.372	Noda et al., 2002
	Striatum	1.045	1.068	0.421	Noda et al., 2002
	Occipital cortex	1.023	1.037	0.418	Noda et al., 2002
	Temporal cortex	0.919	0.940	0.422	Noda et al., 2002
	Frontal cortex	0.778	0.891	0.472	Noda et al., 2002
	Cingulate	0.941	0.956	0.418	Noda et al., 2002
	From plot	1.295	1.437	0.457	Noda et al., 2002

	From plot	0.691	0.575	0.343	Noda et al., 2002
	From plot	0.691	0.627	0.374	Noda et al., 2002
	From plot	0.691	0.627	0.374	Noda et al., 2002
	From plot	1.209	0.914	0.312	Noda et al., 2002
	From plot	1.209	1.254	0.427	Noda et al., 2002
	From plot	1.209	1.359	0.463	Noda et al., 2002
	From plot	1.123	1.097	0.403	Noda et al., 2002
	From plot	1.123	1.123	0.412	Noda et al., 2002
	From plot	1.123	1.254	0.460	Noda et al., 2002
Human	AV difference			0.350	Cohen et al., 2967
	AV difference			0.370	Oshima et al., 2002
	AV difference			0.370	Kety, 1956
	AV difference			0.387	Vernheit et al., 1978
	AV difference			0.382	Laux and Raichle, 1978
	Average, PET			0.410	Derdeyn et al., 2002
	Average, PET			0.400	Raichle et al., 2001
	Average, PET			0.400	Hyder et al., 2016
	Average, PET			0.490	Kaisti et al., 2003
	Average, PET			0.350	Ibaraki et al., 2008
	Average, MRI			0.380	He and Yablonski, 2007
	Average, MRI			0.310	Lee et al., 2017
	Average, MRI			0.360	Jain et al., 2010
	Average, MRI			0.375	Lu et al., 2012
	Average, MRI			0.370	Qin et al., 2011
	Average, MRI			0.350	Xu et al., 2012
Rhesus	AV difference			0.370	Altman et al., 1991

	PET			0.420	Altman et al., 1991
	AV difference			0.430	Laux and Raichle, 1978
	PET			0.420	Noda et al., 2002
Baboo n	PET			0.530	Young et al., 1996
Dog	Radioactive microspheres			0.380	Chen et al., 1984
	MRI			0.300	Chang et al., 2016
	PET			0.418	Weyne et al., 1987
Cat	AV difference			0.380	Hossman et al., 1976
Rat	AV difference			0.316	He et al., 2008
	AV difference			0.420	Johansson and Siesjko, 1975
	AV difference			0.440	Madsen et al., 1998
	AV difference			0.504	Nordstrom and Rehncrona, 1977
	Autoradiography			0.390	Frietch et al., 2000
	AV difference			0.440	Linde et al., 1999
	AV difference			0.430	Schmallbruch et al., 2001
Mouse	MRI			0.350	Zhu et al., 2013
	Autoradiography			0.510	Niwa et al., 2002
	Optical imaging			0.360	Li et al., 2019

Table S2. Data used in Figure 2.

Structure	CMR_{Gluo}, μmol.100mg^{-1}.min^{-1}	Blood flow, ml.100mg^{-1}. min^{-1}	Capillary density, units.mm^{-2}	Ref
amygdala	47			McCulloch et al., 1982

amygdala	56			Kuschinski et al., 1981
amygdala	44			Levant and Pazdernik, 2004
Amygdala	43	97		Nakai et al., 1990
auditory cortex	132		497	McCulloch et al., 1982
auditory cortex	157		497	Kuschinski et al., 1981
Auditory cortex	81	139	497	Nakai et al., 1990
striatum	97		363	McCulloch et al., 1982
Striatum	111		363	Kuschinski et al., 1981
Striatum	88		363	Levant and Pazdernik, 2004
Striatum	69	125	363	Nakai et al., 1990
callosum	33		162	McCulloch et al., 1982
Callosum	42		162	Kuschinski et al., 1981
Callosum	39	63	162	Nakai et al., 1990
Frontal cortex, GM	104		456	McCulloch et al., 1982
Frontal cortex, GM	86		456	Levant and Pazdernik, 2004
Frontal cortex, GM	69	124	456	Nakai et al., 1990
Visual cortex, GM	101		489	McCulloch et al., 1982

Visual cortex, GM	111		489	Kuschinski et al., 1981
Visual cortex, GM	65	99	489	Nakai et al., 1990
Cerebellar WM	34		178	McCulloch et al., 1982
Cerebellar WM	38		178	Kuschinski et al., 1981
Inferior colliculus	191		811	McCulloch et al., 1982
Inferior colliculus	198		811	Kuschinski et al., 1981
Inferior colliculus	171		811	Levant and Pazdernik, 2004
Inferior colliculus	63	173	811	Nakai et al., 1990
Superior colliculus	86		390	McCulloch et al., 1982
Superior colliculus	99		390	Kuschinski et al., 1981
Superior colliculus	73		390	Levant and Pazdernik, 2004
Superior colliculus	63	126	390	Nakai et al., 1990
Lateral geniculate nu.	79		399	McCulloch et al., 1982
Lateral geniculate nu.	92		399	Kuschinski et al., 1981
Dentate gyrus	71		290	McCulloch et al., 1982
Dentate gyrus	53		290	Levant and Pazdernik, 2004
Dentate gyrus, molecular layer	83		363	McCulloch et al., 1982

hippocampus	79		361	Kuschinski et al., 1981
hippocampus	53		361	Levant and Pazdernik, 2004
hippocampus	61	96	361	Nakai et al., 1990
thalamus	88		460	Klein et al., 1986

Table S3. Data used in Figure 3, structured stimulation.

Species	Method	Condition	$\Delta CBF/ CBF_o$	$\Delta CMRO_2/CMRO_{2o}$	OEF	Ref
Human	PET	Achromatic visual stim, @ 1 minute	0.407	0.047	0.300	Mintun et al., 2002
		@ 9 minutes	0.33	0.09	0.330	Mintun et al., 2002
		@ 25 minutes	0.27	0.15	0.360	Mintun et al., 2002
	PET		0.50	0.22	0.330	Marrett and Gjedde, 1997
			0.68	0.25	0.300	Marrett and Gjedde, 1997
			0.32	0.15	0.350	Marrett and Gjedde, 1997
	PET	Stimulation @1 Hz	0.32	0.10	0.330	Vasfaee et al., 1998
		@ 4Hz	0.38	0.15	0.330	Vasfaee et al., 1998
		@ 8Hz	0.41	0.05	0.300	Vasfaee et al., 1998
	Calibrated fMRI	GD 1Hz	0.52	0.26	0.330	Lin et al., 2009
	Calibrated fMRI	GD 4Hz	0.65	0.20	0.290	Lin et al., 2009
	Calibrated fMRI	GD 4Hz	0.45	0.12	0.310	Lin et al., 2009
	PET	Visual stim.	0.34	0.20	0.360	Gjedde and Marrett, 2016
	PET	Visual stim.	0.72	0.23	0.290	Gjedde and Marrett, 2016

	PET	Visual stim.	0.49	0.23	0.330	Gjedde and Marrett, 2016
	Calibrated fMRI		0.42	0.16	0.330	Davis et al., 1998
	fMRI	BW grating lum 20%	0.04	0.03	0.390	Hoge et al., 1999
	fMRI	BW grating lum 40%	0.06	0.03	0.390	Hoge et al., 1999
	fMRI	BW grating lum 60%	0.11	0.06	0.380	Hoge et al., 1999
	fMRI	BW grating lum 80%	0.16	0.08	0.370	Hoge et al., 1999
	fMRI	Red/grey lum 20%	0.03	0.02	0.390	Hoge et al., 1999
	fMRI	Red/grey lum 50%	0.10	0.06	0.390	Hoge et al., 1999
	fMRI	Red/grey lum 75%	0.14	0.08	0.380	Hoge et al., 1999
	fMRI	Red/grey lum 100%	0.26	0.13	0.360	Hoge et al., 1999
	fMRI	4Hz YBRC lum 6.25%	0.11	0.06	0.380	Hoge et al., 1999
	fMRI	4Hz YBRC lum 13%	0.14	0.06	0.370	Hoge et al., 1999
	fMRI	4Hz YBRC lum 19%	0.14	0.08	0.380	Hoge et al., 1999
	fMRI	4Hz YBRC lum 25%	0.17	0.09	0.370	Hoge et al., 1999
	fMRI	8Hz YBRC lum 12.5%	0.27	0.14	0.360	Hoge et al., 1999
	fMRI	8Hz YBRC lum 25%	0.30	0.15	0.350	Hoge et al., 1999
	fMRI	8Hz YBRC lum 38%	0.32	0.16	0.350	Hoge et al., 1999
	fMRI	8Hz YBRC lum 50%	0.43	0.21	0.340	Hoge et al., 1999 [82]

	fMRI	2Hz YRBC 50% saturation	0.30	0.15	0.360	Hoge et al., 1999
	fMRI	4Hz YRBC 50% saturation	0.32	0.18	0.360	Hoge et al., 1999
	fMRI	6Hz YRBC 50% saturation	0.33	0.18	0.350	Hoge et al., 1999
	fMRI	8Hz YRBC 50% saturation	0.43	0.21	0.340	Hoge et al., 1999
	fMRI		0.47	0.23	0.340	Kim et al., 1999
	fMRI		0.78	0.28	0.290	Chiarelli et al., 2007
	fMRI		0.80	0.37	0.300	Ances et al., 2008
	fMRI		0.74	0.32	0.300	Perthen et al., 2008
	fMRI		0.45	0.22	0.340	Griffeth et al., 2011
	fMRI		0.26	0.15	0.360	Liang et al., 2013
	fMRI		0.40	0.19	0.340	Liang et al., 2013
	fMRI		0.41	0.18	0.340	Liang et al., 2013
	fMRI		0.55	0.24	0.320	Liang et al., 2013
	fMRI		0.21	0.15	0.380	Germuska et al., 2019
	fMRI	Low contrast checkerboard	0.18	0.07	0.360	Griffeth et al., 2015
	fMRI	High contrast checkerboard	0.40	0.17	0.330	Griffeth et al., 2015
	fMRI	Natural scene	0.29	0.12	0.350	Griffeth et al., 2015
	fMRI	1Hz	0.45	0.15	0.320	Zhang et al., 2019
	fMRI	4Hz	0.49	0.18	0.320	Zhang et al., 2019
Rat	FDG/14C glucose	seizure	1.12	0.51	0.280	Ackerman and Lear, 1989
	FDG/14C glucose	seizure	0.18	0.11	0.380	Ackerman and Lear, 1989
	FDG/14C glucose	Visual stim 8Hz	0.55	0.18	0.300	Collins et al., 1987

	FDG/14C glucose	Visual stim 16Hz	0.60	0.28	0.300	Collins et al., 1987
	FDG/14C glucose	auditory	0.47	0.17	0.320	Cruz et al., 2007
	FDG	mixed	0.66	0.34	0.320	Dienel et al., 2007
	14C glucose	mixed	0.72	0.41	0.330	Dienel et al., 2002
	AV difference	mixed	0.72	0.30	0.330	Madsen et al., 1999

Table S4. Additional data used in Figure 4.

Species	Structure	CBF/CBF_o	$CMRO_2/CMRO_{2o}$	OEF	Ref
Human	AV difference	0.81	0.75	0.370	Madsen et al., 1991
	AV difference	0.89	0.84	0.380	Boyle et al., 1994
	PET	0.90	0.92	0.400	Takahashi, 1989
	fMRI	0.81	0.78	0.390	Uludag et al., 2004
	fMRI	0.90	0.90	0.400	Koush & Hyder, unpublished

All other values of CBF/CBF_o and $CMRO_2/CMRO_{2o}$ correspond to 1 + the values in Table S2.
